# The impact of bariatric surgery on assisted reproductive technology outcomes: a systematic review protocol

**DOI:** 10.1186/s13643-021-01870-8

**Published:** 2022-01-03

**Authors:** Kameela Alibhai, Isabella Churchill, Tannys Vause, Heather Anne Lochnan

**Affiliations:** 1 Faculty of Medicine, University of Ottawa, 501 Smyth Rd, Ottawa, Ontario K1H 8L6 Canada; 2 Department of Obstetrics and Gynecology, Faculty of Medicine, University of Ottawa, Ottawa, Canada; 3 Department of Medicine, The Ottawa Hospital, University of Ottawa, Ottawa, Canada

**Keywords:** Assisted reproductive technology, Fertility, In vitro fertilization, Intracytoplasmic sperm injection, Bariatric, Weight loss surgery, Obesity, Body mass index

## Abstract

**Background:**

Individuals with obesity are at higher risk of experiencing complications during their pregnancy and may also experience infertility, requiring assisted reproductive technologies (ART) to conceive. The current body of literature demonstrates that bariatric surgery decreases an individual’s risk of developing a variety of obesity-related obstetrical conditions during and after pregnancy. However, the effects of bariatric surgery on ART outcomes are not well understood. Therefore, the paucity in the literature warrants a need to determine these effects.

**Methods:**

We will search electronic databases, including MEDLINE, Embase, Scopus, and Cochrane Central Register of Controlled Trials (CENTRAL), as well as the gray literature and the reference lists of included articles. We will screen all studies published between January 1978 and the present day that explore the impact of bariatric surgery on ART outcomes for women and men. We will include observational studies. Two independent reviewers will assess the studies for inclusion and extract data for each article. The main outcome that will be analyzed is live birth rate. Secondary outcomes such as time to conception, number of rounds of ART, type of bariatric surgery, and length of time between bariatric surgery and initiation of ART will also be recorded. Risk of bias will be conducted using the National Institutes of Health Study Quality Assessment Tools. A random effects model will be used to account for statistical analysis and results will be pooled with forest plots. In the event of statistical and reporting heterogeneity, we will provide a qualitative synthesis and narrative review of the results.

**Discussion:**

This review will provide information on the outcomes of ART following bariatric surgery and may help healthcare professionals make informed decisions about the length of time between bariatric surgery and initiation of ART. The study findings may be of interest to various stakeholders including patients, bariatric surgeons, obstetricians, and gynecologists, and those who specialize in obesity medicine and reproductive endocrinology and infertility. We plan to disseminate our findings through presentations, publications, and social media releases to individuals who are navigating infertility and are interested in undergoing or have undergone bariatric surgery, healthcare professionals, policymakers, and researchers.

**Systematic review registration:**

PROSPERO CRD42021252561

## Introduction

### Rationale

Obesity, which is the accumulation of an abnormal or excessive amount of adipose tissue, is a global epidemic that affects over 650 million adults worldwide [1]. The WHO defines obesity according to body mass index (BMI), which is a measure of fat calculated by dividing an individual’s weight (kg) by the square of their height (m^2^) [1]. Obesity is a chronic illness that is caused by a combination of metabolic, behavioral, genetic, and environmental factors. Obesity increases one’s risk of developing health complications and long-term diseases, such as cardiovascular disease and cancer [1, 2].

The prevalence of obesity among individuals of reproductive age around the world is reported to be as high as 30% [3–5]. One study has estimated that the prevalence of overweight and obese individuals in developed countries is proposed to reach levels of 89% and 85% in males and females, respectively, accounting for 3.5 million people [6]. Furthermore, it is noted that approximately 40‑65% of females in their reproductive years will be considered obese [6]. Females who have obesity during pregnancy are at higher risk of experiencing miscarriages, gestational diabetes mellitus, preeclampsia, complications with anesthesia, and post-partum hemorrhage [7, 8]. In males, obesity has been related to changes in sperm count, motility, and morphology as well as hormonal disturbances that decrease total and free levels of testosterone [9]. Accordingly, they may also require assisted reproductive technologies (ART) to conceive [3, 7], with studies reporting an increasing trend in the use of ART within developed countries [10–12]. In response to gonadal stimulation performed as part of ART, individuals with obesity may demonstrate a suboptimal response to gonadotropins, present with fewer follicles on ultrasound and fewer oocytes upon retrieval [7, 13]. Due to these suboptimal outcomes, some countries have imposed limitations that prevent people with obesity from being able to access ART or receive funding to offset the costs [14].

Bariatric surgery is one of the most effective and sustainable weight loss strategies for those of reproductive age. Approximately 80% of all patients undergoing this procedure identified as women and 56% of them were between the age of 30 and 46 years [15]. Although bariatric surgery may lead to vitamin deficiencies and gastrointestinal symptoms, individuals can successfully become pregnant after undergoing bariatric surgery [13]. The current body of literature demonstrates that bariatric surgery decreases an individual’s risk of developing a variety of obesity-related obstetrical conditions during and after pregnancy [16]. However, the effects of bariatric surgery on ART outcomes are not well understood. Examining the effects of bariatric surgery on ART outcomes is needed, especially considering a high number of individuals who undergo this procedure are of reproductive age [17]. Systematic reviews have analyzed the effects of bariatric surgery on various fertility outcomes; however, they have not included ART [18, 19]. Additionally, another review conducted in 2014 examined the effects of weight loss in obese women undergoing fertility treatment [20]. Within the past 8 years, several new studies, including retrospective cohort studies and randomized controlled trials (RCT), have been carried out. This highlights the value of conducting a systematic review on the effects of bariatric surgery on ART outcomes that will include an analysis of the current, more robust body of literature.

### Objective

The aim of this systematic review is to identify the effects of bariatric surgery on ART outcomes. For the purposes of this study, ART will encompass in vitro fertilization (IVF) and intracytoplasmic sperm injection (ICSI) procedures. Accordingly, our review aims to answer the following research question: does bariatric surgery have any impact on ART outcomes?

## Methods

This study protocol was developed and reported based on the Preferred Reporting Items for Systematic Reviews and Meta-Analysis (PRISMA) Guidelines [21], and was submitted for registration with the International Prospective Register of Systematic Reviews (PROSPERO), an open access online database of systematic review protocols, in May of 2021.

### Eligibility criteria

Any observational study—prospective and retrospective comparative cohort studies, case control studies, case series, and case reports—that examine the influence of bariatric surgery on ART outcomes will be eligible for inclusion. Cross sectional studies will be excluded. Studies will be required to have taken place in a developed country. Participants will be required to have used ART following their bariatric surgery. We will include human studies written in English from 1978, the year the first IVF baby was born [22], to present day. We will exclude animal studies; studies with pediatric populations (participants < 18 years old); and studies with populations restricted to specific diseases, conditions, or metabolic disorders. Dissertations, conference proceedings, and abstracts will also be excluded.

Eligibility criteria will follow the Population, Intervention, Comparison, Outcomes, Timeframe, Study framework [23, 24].

#### Population

Studies with women and men participants of reproductive age (≥ 18 years old), who have a prior history of bariatric surgery, and are undergoing ART will be included. Studies which exclusively investigated selected populations with specific disease conditions (e.g., individuals with polycystic ovary syndrome or testicular cancer) or metabolic disorders (e.g., diabetes or Gaucher disease) will be excluded, as well as studies that do not use body mass index (BMI) as a parameter for obesity (e.g., waist to hip ratio and body weight). According to WHO, obesity is defined as abnormal or excessive fat accumulation that presents a risk to health [25]. Obesity will be defined based on the participant’s BMI prior to their bariatric surgery (BMI is over 40, or if BMI exceeds 35 with severe comorbidities) [26, 27]. Participant’s BMI should have been measured following their bariatric surgery and/or prior to starting fertility treatment; however, this data may not be recorded in all included studies.

#### Interventions

Studies examining ART interventions, specifically IVF, ICSI, and fertility preservation, will be deemed eligible for inclusion.

#### Comparator

No comparator group will be used.

#### Outcomes

Studies examining intrauterine, intracervical, or artificial insemination will be excluded as these procedures only handle sperm and are not considered types of ART for the purposes of this study [28]. Studies investigating the effect of bariatric surgery on natural cycle conceptions will also be excluded. Studies with available data on live birth rate will be eligible for inclusion.

### Information sources

Our search strategy will employ medical subject headings (MeSH) terms as well as keywords related to fertility and bariatric surgery. We will search MEDLINE, EMBASE, SCOPUS, and the Cochrane Central Register of Controlled Trials (CENTRAL). PROSPERO, OSF Pre-print, and SCOPUS will be searched to identify any ongoing or recently completed systematic reviews. For completeness, we will search the gray literature (e.g., clinicaltrial.gov, the International Clinical Trials Registry Platform (ICTRP), and OpenGrey) as well as the reference lists of included studies and of relevant reviews identified through the search. A preliminary search was carried out on May 28, 2021.

### Search strategy

The specific search strategy employed has been developed in consultation with a medical librarian with expertise in systematic review searching. Our search will include the terms “bariatric surgery,” “assisted reproductive techniques,” “in vitro fertilization,” and “obesity.” A draft of the MEDLINE search strategy is included (Table 1 in Appendix). After the MEDLINE search strategy has been finalized, it will be formatted for other databases of interest and will employ database-specific vocabulary when applicable. The search will be performed by the first reviewer (KA).

### Selection process

The results from this systematic literature search will be complied into EndNote, a reference manager software. Duplicates will be removed. The articles will then be uploaded to the Covidence platform, a web-based review software, which identifies and removes any further duplicates, streamlines screening of citations, and facilitates the resolution of conflicts between reviewers. Keywords and MeSH terms will be highlighted on the platform to facilitate screening. Citation abstracts and full-text articles will be uploaded to Covidence. Prior to the formal screening process, team members will be provided with training on how to use Covidence and receive background information on this research topic. A pilot of the screening will be conducted to ensure a high agreement among raters.

Two reviewers (KA and IC) will first independently screen title and abstracts in duplicate according to defined eligibility criteria. Potentially relevant articles will then be advanced to full-text review, which will be completed by two reviewers (KA and IC). The reasons for exclusion of studies will be recorded in Covidence. Differences will be resolved through discussion between the two reviewers. When reviewers disagree, the article will be referred to the senior author (HL).

### Data extraction process

A standardized data extraction form will be created by one of the reviewers (IC). Two reviewers (KA and IC) will independently extract the data into a standardized Excel spread sheet. Reviewers will resolve disagreements through discussions, and any remaining disagreements will be resolved in consultation with the senior author (HL).

### Data items prioritization

The following demographic and study data will be extracted from all included studies: study authors, year of study, study setting (e.g., country/city), study design, and study aim. Participant demographics including age; BMI and/or weight loss; diagnosis; antral follicle count (AFC) on ultrasound; anti-Mullerian hormone; and day 3 follicle stimulating hormone levels, supplements, and comorbidities will also be extracted.

#### Primary outcome

The primary outcome for this systematic review was chosen based on Reproductive Endocrinology and Infertility Committee’s guidelines [29]. The main outcome that will be analyzed is *cumulative live birth rate* defined as a pregnancy that results in a baby, irrespective of the duration of pregnancy, which, after such separation or extraction, shows evidence of life [30].

#### Secondary outcomes

Additional outcomes about the intervention will include time to conception, number of rounds of ART, type of bariatric surgery, and length of time between bariatric surgery and initiation of ART. The following sex-specific secondary outcomes will also be extracted:

**Table:** 

Female outcomes	Male outcomes
Fertilization rate Implantation rate Miscarriage rate Total number of mature oocytes Number of mature oocytes Complications Pre-term birth Mode of delivery Gestational weight gain Intrauterine growth restrictions Total gonadotropin dose	Sperm motility Sperm morphology Total sperm count Sperm concentration Mature spermatozoa

### Risk of bias

The risk of bias for each of the included studies will be assessed by two reviewers independently (KA and IC). A quality assessment will be conducted on each of the included studies using the National Institutes of Health (NIH) Study Quality Assessment Tools [31]. Several NIH quality assessment tools exist to assess a wide variety of study designs. As such, the tool that will be utilized will be specific to the study design of the article being reviewed. We will summarize the risk of bias within studies using the tools appropriate to the study design. Differences will be resolved through discussion between the two reviewers or in consultation with a third reviewer (HL) when necessary.

### Data analysis

We expect that there will be limited scope for meta-analysis due to the range of different types of bariatric surgeries, ART interventions, and fertility outcomes measured across studies. However, if the data will allow, we plan to pool outcomes in a meta-analysis. Dichotomous data will be recorded as a rate or count and continuous data will be recorded as the mean value or proportion. Statistical heterogeneity will be assessed using *I*^2^. Substantial heterogeneity will be defined as *I*^2^ > 75% [32]. The R Software (R Studio, 2013, Vienna, Austria) will be used to pool primary and secondary outcomes in meta-analyses using forest plots. The pooled data will be computed using the DerSimonian-Laird method, under a random-effect model, and the 95% confidence interval (CI) will be estimated for the dichotomous outcomes (live birth or no live birth) [32]. If possible, a meta-regression will be performed for secondary outcomes.

The following subgroup analyses plan to be investigated: (1) timeline between bariatric surgery and ART; (2) categories of BMI (obesity class 1, class 2, and class 3); (3) type of ART intervention (IVF vs ICSI); (4) age; and (5) sex. If appropriate, a sensitivity analysis will be conducted based on the quality of studies (“good” vs “fair” vs “poor”) to determine the robustness of the results.

Variable length of follow-up can lead to unit of analysis error. As such, separate analyses based on previously defined length of follow-up will be carried out. In the case of missingness of relevant data, authors will be contacted to request sufficient information. If this is not possible, missing data will be accounted for in the risk of bias assessment and explored in a sensitivity analysis.

In the likely circumstance that substantial heterogeneity prohibits the appropriate use of a meta-analysis, we will provide a qualitative synthesis of the findings from the included studies, structured around type of assisted reproductive technology involved, fertility outcomes, significant improvement in quantity and *p* value, and effect size. We will provide summaries of study characteristics, variable measures, data analysis models, significant study findings, and reported effect sizes.

### Reporting bias and certainty assessment

If possible, publication bias will be assessed with a funnel plot and the quality of evidence will be assessed using the Grading of Recommendations Assessment, Development and Evaluation (GRADE) approach for both primary and secondary outcomes. The four levels of quality (high, moderate, low, and very low) will be used to assess the evidence related to the primary outcome. GRADE profiler (GRADEPRO) [33] will be used to import data from Review Manager 5.2.4 to create a “summary of findings” table.

## Discussion

There is a lack of agreement among healthcare professionals on the effectiveness and timing of ART following bariatric surgery. As such, this review aims to synthesize the findings of studies addressing the effects of bariatric surgery on fertility outcomes in individuals receiving IVF or ICSI treatments. The review will be based on published studies from 1978, the year the first IVF baby was born, to the present day and will allow us to assess the quality of the published studies as well as analyze outcome data. We anticipate that there will be a limited number of high-quality trials on this subject matter and that extracted data will be heterogeneous, which may limit our quantitative analyses. The study findings may be of interest to various stakeholders including patients, bariatric surgeons, obstetricians, and gynecologists, as well as obesity medicine and infertility specialists. Furthermore, our findings may help healthcare professionals make informed decisions about the length of time between bariatric surgery and initiation of ART. This review may also reveal gaps in the literature and highlight additional research questions related to the use of ART among individuals with a history of bariatric surgery.

Previous meta-analyses have shown that following in vitro fertilization (IVF), obese individuals are significantly less likely to achieve clinical pregnancy and give birth to a live infant [34, 35] and significantly more likely to experience various pregnancy complications [36] and to develop gestational diabetes [19]. Although it is known that weight loss improves both fertility and pregnancy outcomes, we hope to use the results from this review to draw conclusions on the impact of bariatric surgery on ART outcomes. We also hope to determine various sex-specific outcomes that may also need to be further researched as bariatric surgery likely affects ART outcomes in females and males differently. Finally, future research should identify the optimal type of bariatric surgery and time when ART should be initiated following surgery. This will allow fertility clinicians to counsel patients about potential complications and the most appropriate time to initiate ART.

We plan to disseminate our findings to individuals who are navigating infertility and are interested in undergoing or have undergone bariatric surgery, healthcare professionals, policymakers, and researchers working in obesity and/or infertility medicine. Our study team intends to prepare a manuscript for publication in a peer-reviewed journal and present our findings at international and national conferences in the domain of reproductive endocrinology and infertility. We also plan to circulate our findings (in lay terms) through social media (e.g., Twitter and LinkedIn) to ensure widespread dissemination among patients and professionals alike.

## Data Availability

N/A

## Appendix

**Table 1:** MEDLINE search (OVID platform) performed on May 28, 2021

Search #	Search Terms	Results
1.	exp Bariatric Surgery/	28385
2.	((bariatric or metabolic) adj3 (surger* or procedure?)).ti,ab.	20156
3.	(stomach adj3 stapl*).ti,ab.	66
4.	(restrictive adj3 surger*).ti,ab.	340
5.	(gastric adj3 surger*).ti,ab.	8965
6.	gastroplast*.ti,ab.	2113
7.	anti?obesity surger*.ti,ab.	22
8.	(antiobesity adj3 surger*).ti,ab.	30
9.	exp gastric bypass/	10504
10.	exp jejunoileal bypass/	600
11.	(jejuno?ileal adj3 bypass).ti,ab.	844
12.	(gastrointestinal adj3 (surg$ or diversion$)).ti,ab.	5658
13.	exp biliopancreatic diversion/	1044
14.	(bilio?pancreatic adj3 (diversion$ or bypass)).ti,ab.	1212
15.	gastric adj3 band$).ti,ab.	4038
16.	(silicon adj3 band$).ti,ab.	268
17.	exp gastroenterostomy/	13954
18.	gastrectomy.ti,ab.	30148
19.	((gastric or intestinal) adj3 bypass).ti,ab.	13029
20.	exp gastroplasty/	4410
21.	LAGB.ti,ab.	1173
22.	(lap adj3 band$).ti,ab.	280
23.	(malabsorptive adj3 (surger* or procedure?)).ti,ab.	333
24.	"Roux-en-Y".ti,ab.	1
25.	(duodenal adj3 switch$).ti,ab.	836
26.	1 or 2 or 3 or 4 or 5 or 6 or 7 or 8 or 9 or 10 or 11 or 12 or 13 or 14 or 15 or 16 or 17 or 18 or 19 or 20 or 21 or 22 or 23 or 24 or 25	75398
27.	Reproductive Techniques, Assisted/	9973
28.	(assisted reproduct*).ti,ab.	16222
29.	exp Fertilization in Vitro/	37070
30.	((in vitro adj2 fertili?ation) or IVF).ti,ab.	36848
31.	exp sperm injection, intracytoplasmic/	6984
32.	((intracytoplasmic sperm adj2 injection?) or ICSI).ti,ab.	11393
33.	exp embryo transfer/ (	16729
34.	(embryo adj2 transfer).ti,ab.	12445
35.	27 or 28 or 29 or 30 or 31 or 32 or 33 or 34	71108
36.	26 and 35	48
37.	limit 37 to yr="1978 -Current"	48

Total = 48 article results

## Abbreviations

ART: Assisted reproductive technology
BMI: Body mass index
GRADE: Grading of Recommendations Assessment, Development and Evaluation
ICSI: Intracytoplasmic sperm injection
IVF: In vitro fertilization
OR: Odds ratio
PRISMA: Preferred Reporting in Systematic Reviews and Meta-Analysis
PROSPERO: International Prospective Register of Systematic Reviews
RevMan: Review Manager
RR: Risk ratio
